# Germ cell-specific Atg7 knockout results in primary ovarian insufficiency in female mice

**DOI:** 10.1038/cddis.2014.559

**Published:** 2015-01-15

**Authors:** Z-H Song, H-Y Yu, P Wang, G-K Mao, W-X Liu, M-N Li, H-N Wang, Y-L Shang, C Liu, Z-L Xu, Q-Y Sun, W Li

**Affiliations:** 1 State Key Laboratory of Reproductive Biology, Institute of Zoology, Chinese Academy of Sciences, Beijing, PR China; 2 University of Chinese Academy of Sciences, Beijing, PR China

## Abstract

Primary ovarian insufficiency (POI) is a common cause of infertility in around 1–2% of women aged <40 years. However, the mechanisms that cause POI are still poorly understood. Here we showed that germ cell-specific knockout of an essential autophagy induction gene *Atg7* led to subfertility in female mice. The subfertility of *Atg7* deletion females was caused by severe ovarian follicle loss, which is very similar to human POI patients. Further investigation revealed that germ cell-specific *Atg7* knockout resulted in germ cell over-loss at the neonatal transition period. In addition, our *in vitro* studies also demonstrated that autophagy could protect oocytes from over-loss by apoptosis in neonatal ovaries under the starvation condition. Taken together, our results uncover a new role for autophagy in the regulation of ovarian primordial follicle reservation and hint that autophagy-related genes might be potential pathogenic genes to POI of women.

Primary ovarian insufficiency (POI), also known as premature ovarian failure (POF), is an ovarian defect characterized by the premature depletion of ovarian follicles before the age of 40 years. POI is a common cause of infertility in women, affecting 1–2% of individuals aged <40 years and 0.1% of individuals aged <30 years.^1^ Potential etiologies for POI are highly heterogeneous, which include iatrogenic, infectious, autoimmune, metabolic, chromosomal and genetic factors.^2^ At present, about 25% of all forms of POF can be classified as iatrogenic and are related to cancer treatment, but >50% of the cases remain idiopathic. Though the pathogenic mechanism remains unexplained in the majority of the cases, several observations support a prevalent role of genetic mechanisms in the pathogenesis of idiopathic POI. It has been reported that mutations in FMR1, BMP-15, GDF-9, FOCL2, FSHR, LHR, INHA, GALT and AIRE are associated with POI.^3, 4, 5, 6, 7, 8, 9, 10, 11, 12, 13^ The genetic information of POI is very useful for family counseling, because it can predict the female relatives who may be at higher risk for POI and fertility loss in young age. The female carriers will be able to plan their conception before ovarian failure occurs. This requirement is becoming more and more important, because women nowadays tend to conceive ever more frequently in their thirties and forties,^10^ when the risk of POI in the general population is about 1–2%. However, still few genes could be identified that can explain a substantial proportion of the cases of POI.

An important phenotype of POI is infertility, thus POI patients do not have large family histories, and therefore are difficult to study using traditional genetic methods, such as linkage analysis. Animal models of POI have been successfully used to identify candidate genes in this disease. The disruption of meiosis-specific genes, Bcl-2 family apoptotic-related genes, Pten-PI3K-Akt-Foxo3 pathway and Tsc1/2-mTOR signaling pathway result in POI-like phenotype in mice.^14, 15, 16, 17^ However, as a complex disorder, the genetic etiologies of POI still need to be further investigated to better understand the underlying molecular mechanisms.

Macroautophagy (hereafter referred to as autophagy) is the primary intracellular catabolic mechanism for degrading and recycling long-lived proteins and organelles, which is evolutionarily conserved from yeast to mammals.^18^ During autophagy, isolation membrane enwraps parts of the cytoplasm and intracellular organelles, and fuse with each other forming a double membrane structure, known as the autophagosome. Then the outer membrane of the autophagosome fuses with the lysosome to form autolysosome, in which the cytoplasm-derived materials are degraded by resident hydrolases.^19^ The primary function of autophagy is to allow cells or organisms to survive nutrient starvation conditions by recycling either proteins or other cellular components. This process is important for cells to adapt their metabolism to starvation caused by decreased extracellular nutrients or by decreased intracellular metabolite concentrations. In addition to nutrient supply and adaptation to stress conditions, a number of observations have revealed that autophagy also functions in many physiological processes in mammalian systems, such as cell death, antiaging mechanisms, innate immunity, development and tumor suppression.^20, 21, 22, 23, 24, 25^

From the discovery of the molecular mechanism underlying autophagy, it was found that autophagy is required for the reproductive process in budding yeast.^26^ In mammals, fertilization induces massive autophagy to degrade maternal proteins and messenger RNAs, and autophagy functions as a major nutrient-providing system for embryos before their implantation.^27^ Our recent work indicates that autophagy is required for acrosome biogenesis during spermatogenesis in mice, thus essential to male fertility.^24^ However, whether autophagy is involved in female gametogenesis or not is still unknown. Here, we showed that germ cell-specific knockout of an essential autophagy induction gene *Atg7* led to POI in female mice, and the numbers of the oocytes and follicles were significantly declined in the adult mutant mice. Further investigation revealed that autophagy protected oocytes over-loss during the neonatal transition period. Our results suggest that autophagy-related genes might be pathogenic genes to POI.

## Results

### Generation of germ cell-specific *Atg7* knockout female mice

**Figure 1:**
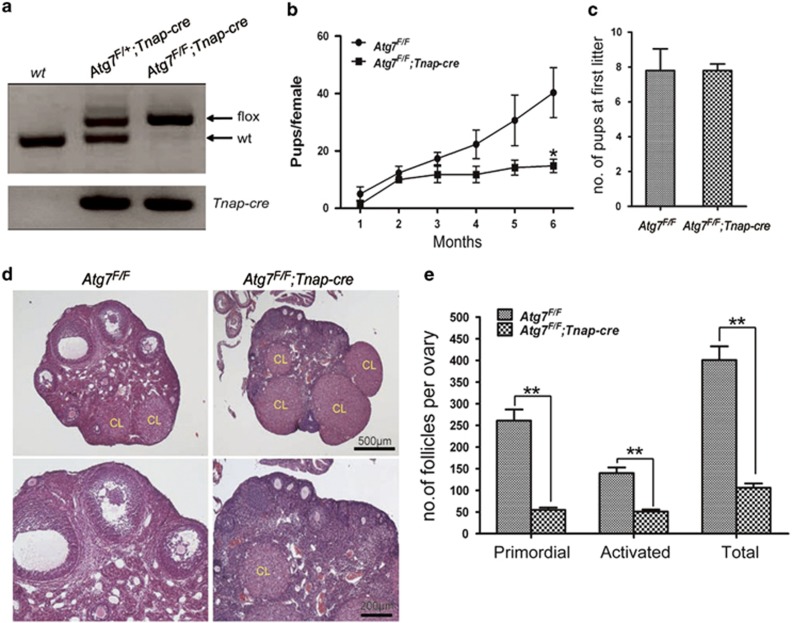
Disruption of Atg7 in oocytes led to female subfertility with follicle over-loss. (**a**) PCR confirmation of the *Atg7*^*F/F*^*;Tnap-cre* mice. (**b**) Subfertility of the female *Atg7*^*F/F*^*;Tnap-cre* mice. Both *Atg7*^*F/F*^ (*n*=6) and *Atg7*^*F/F*^*;Tnap-cre* (*n*=5) females were mated with wild-type males; continuous breeding assessment showed the cumulative number of progeny per female mouse. Asterisk indicates statistically significant difference in comparison with *Atg7*^*F/F*^. (**c**) The first litter size of *Atg7*^*F/F*^ (*n*=6) and *Atg7*^*F/F*^*;Tnap-cre* (*n*=5) females. (**d**) Representative hematoxylin and eosin staining of ovaries from 6-month-old mice of each genotype. CL, corpus luteum. (**e**) Quantification of the follicle numbers in ovaries of *Atg7*^*F/F*^ (*n*=6) and *Atg7*^*F/F*^*;Tnap-cre* (*n*=5) mice at 6 months of age. Data are presented as the mean±S.E.M. *P<0.05, **P<0.01

The core molecular machinery of autophagy was driven by two ubiquitin-like conjugation systems, which are required for the autophagosome formation. Autophagy-related gene 7 (Atg7) is the homologous of the ubiquitin-activating enzyme E1, which is essential to both of these two conjugation systems.^28^ Because *Atg7*-deficient mice died within 1 day after birth,^29^ we cannot study its role in female reproduction directly, thus we generated a germ cell-specific *Atg7* knockout mouse line by crossing *Atg7*^*F/F*^mice with transgenic mice expressing Tnap promoter-mediated Cre recombinase. In *Tnap-Cre* mice, Cre is expressed in primordial germ cells,^30^ and its expression resulted in germ cell-specific Atg7 knockout mice hereafter called *Atg7*^*F/F*^*;Tnap-cre*. Figure 1a shows the genotyping of *Atg7*^*F/F*^*;Tnap-cre* mice.

### Germ cell-specific *Atg7* knockout causes subfertility due to follicles over-loss

To test the female fertility after germ cell-specific Atg7 knockout, a breeding assay was conducted by mating *Atg7*^*F/F*^ or *Atg7*^*F/F*^*;Tnap-cre* female mice with *Atg7*^*F/F*^ males for 6 months. As shown in Figure 1b, female *Atg7*^*F/F*^*;Tnap-cre* mice were severely subfertile and gave birth to about 63% fewer pups than *Atg7*^*F/F*^ mice (14.80±2.332 *versus* 40.33±8.686, *P*<0.05). Interestingly, all the five randomly selected *Atg7*^*F/F*^*;Tnap-cre* females were fertile at their first conception, and they gave birth 7–8 pups at their first litter, which was similar to the *Atg7*^*F/F*^ mice (Figure 1c). However, in the subsequent assessment, they suddenly gave significantly less pups per litter or became completely sterile. This phenotype was very similar to POI in human patients.

To explore the mechanism underlying the reproductive defects caused by *Atg7* knockout, we further compared follicular development in 6-month-old *Atg7*^*F/F*^*;Tnap-cre* with that in *Atg7*^*F/F*^ mice. As shown in Figure 1d, although primordial, activated follicles and corpus luteum were found in 6-month ovaries of *Atg7*^*F/F*^ and *Atg7*^*F/F*^*;Tnap-cre* mice, the number of the follicles appeared to be reduced in the *Atg7*^*F/F*^*;Tnap-cre* mice compared with the *Atg7*^*F/F*^mice. To determine whether the deletion of Atg7 affected follicular development, we performed accurate counts of primordial and activated follicles. As shown in Figure 1e, the total number of the follicles was seriously reduced in the *Atg7*^*F/F*^*;Tnap-cre* mice compared with the *Atg7*^*F/F*^mice (106±9.866 *versus* 401±31.75, *P*<0.05); the primordial follicles (55±5.196) and activated follicles (51±4.726) population in the *Atg7*^*F/F*^*;Tnap-cre* mice had declined by 79% and 64%, respectively, compared with that in the control group (261.0±25.7, 140±12.86, *P*<0.05). These histological results revealed that the number of follicles was severely affected by *Atg7* deletion, especially for the primordial follicles, suggesting that the subfertility in female *Atg7* knockout mice might be caused by the germ cell over-loss.

### Germ cell-specific *Atg7* knockout leads to oocyte over-loss during neonatal transition

**Figure 2:**
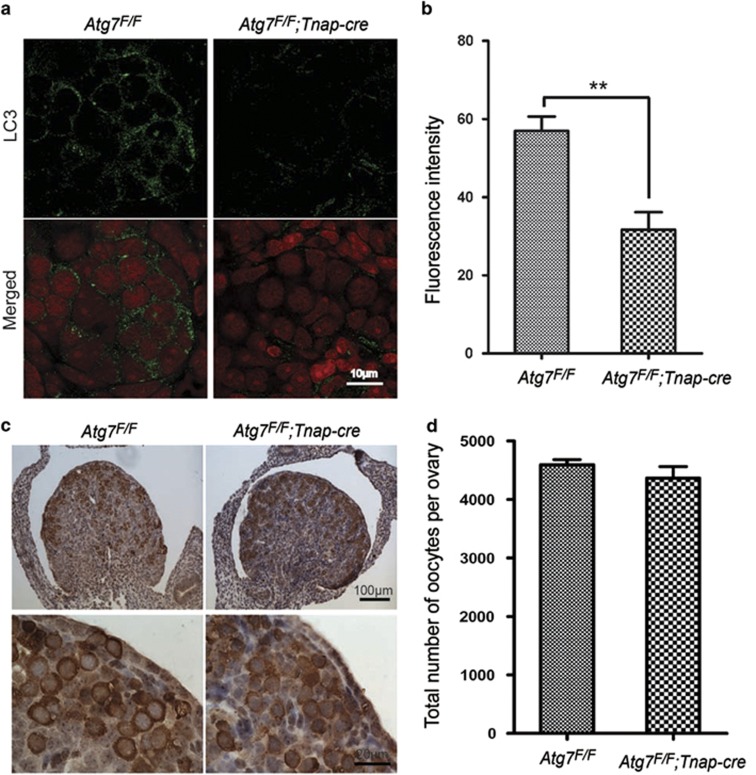
Atg7 knockout did not affect oocytes number in fetal ovaries on 17.5 dpc. (**a**) Autophagy was severely disrupted in 17.5 dpc *Atg7*^*F/F*^*;Tnap-cre* mice (*n*=3) ovaries by immunostaining of LC3. LC3 (in green), nuclear (in red). (**b**) Quantification of LC3 fluorescent intensity in panel (**a**). (**c**) Morphological analysis of 17.5 dpc fetal ovaries of *Atg7*^*F/F*^*;Tnap-cre* mice and the control. Germ cell was indicated by immunohistochemical detection of MVH. (**d**) Quantification of oocytes number in 17.5 dpc fetal ovaries of *Atg7*^*F/F*^*;Tnap-cre* mice (*n*=3) and the control (*n*=3). Data are presented as the mean±S.E.M. **P<0.01

**Figure 3:**
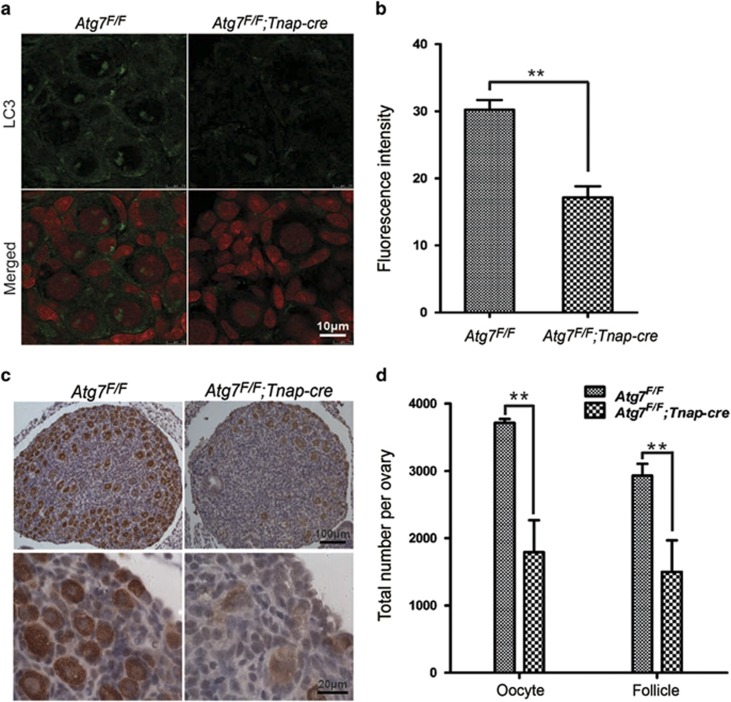
Oocytes and follicles over-loss in 3-day-old Atg7 knockout mice ovaries. (**a**) Autophagy was severely disrupted in 3-day-old *Atg7*^*F/F*^*;Tnap-cre* mice (*n*=3) ovaries by immunostaining of LC3. LC3 (in green), nucleus (in red). (**b**) Quantification of LC3 fluorescent intensity in panel (**a**). (**c**) Morphological analysis of 3-day-old ovaries after Atg7 knockout. Germ cell was indicated by immunohistochemical detection of MVH. (**d**) Quantification of oocyte and follicle numbers in 3-day-old mice ovaries of *Atg7*^*F/F*^*;Tnap-cre* mice (*n*=3) and the control (*n*=3). Oocytes at least surrounded by three cells were counted as follicles. Data are presented as the mean±S.E.M. **P<0.01

In mammals, the profound cell loss occurs at birth in the ovary, which precedes the establishment of a fixed follicle reserve that is progressively depleted during the reproductive lifespan. To clarify the key stage for oocyte over-loss after *Atg7* deletion, we examined the germ cell numbers by immunohistochemistry using a germ cell molecular marker mouse vasa homolog (MVH) in prenatal and postnatal *Atg7*^*F/F*^*;Tnap-cre* ovaries. In 17.5-dpc (day postcoitum) ovaries, although the expression of an autophagy marker microtubule-associated protein 1 light chain 3 (LC3)^31^ decreased very significantly in *Atg7* knockout ovary compared with that in the *Atg7*^*F/F*^, no apparent difference was found in the morphological analysis between *Atg7*^*F/F*^*;Tnap-cre* and *Atg7*^*F/F*^ (Figure 2c). The number of oocytes also showed no significant difference between *Atg7*^*F/F*^*;Tnap-cre* (4591±88.22) and *Atg7*^*F/F*^ (4365±195.7) (Figure 2d). Therefore, we further compared follicular development in 3-day-old *Atg7*^*F/F*^*;Tnap-cre* mice to that in *Atg7*^*F/F*^mice. As shown in Figures 3a and b, the expression of LC3 in *Atg7* knockout ovary was clearly less than that in the control ovary. Different from those 17.5-dpc ovaries, the numbers of the oocytes (1792±477.2) and follicles (1498±470.7) in the *Atg7*^*F/F*^*;Tnap-cre* ovaries were significantly reduced compared with the *Atg7*^*F/F*^ (3716±102.6, 2930±175.2, respectively), 52% oocytes and 49% follicles were lost after *Atg7* knockout in germ cells (Figures 3c and d). These results suggested that Atg7 might be necessary for germ cells survival during the neonatal transition.

### Autophagy is induced in the ovary of normal neonatal mice

**Figure 4:**
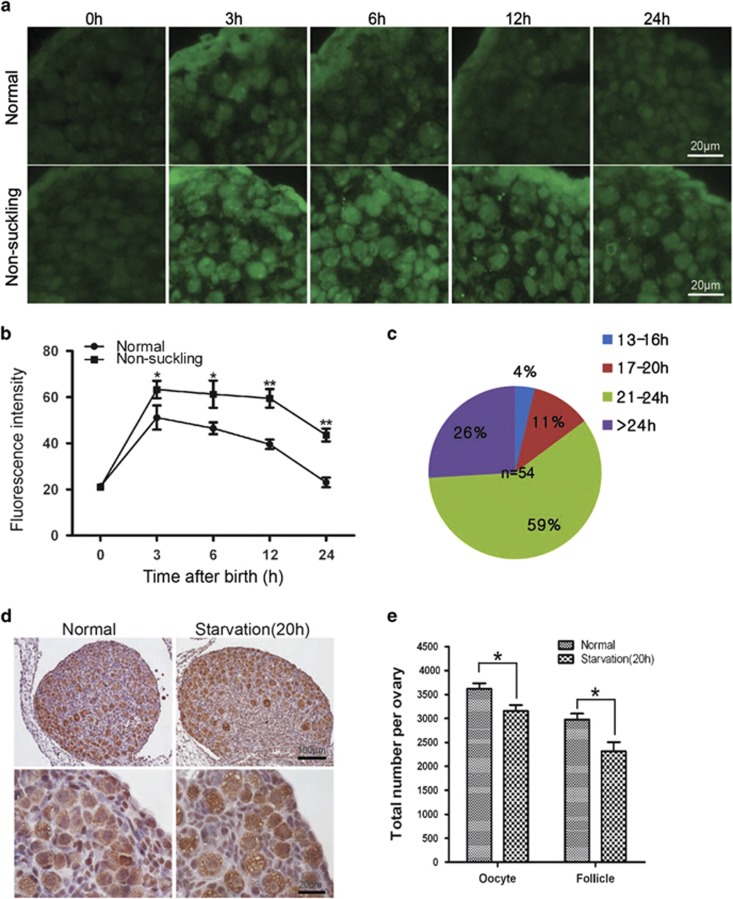
Autophagy was induced in mice ovary during neonatal transition. (**a**) Autophagy induction in the neonatal ovary indicated by immunostaining of LC3 (in green). In the non-suckling groups, mice were separated from the mother immediately after birth. Ovaries were isolated from mice at 0, 3, 6, 12 and 24 h, immediately fixed, cryosectioned, and analyzed by fluorescence microscopy. (**b**) Quantification of LC3 fluorescent intensity in panel (**a**). Asterisks indicate statistically significant difference in comparison with the normal group. (**c**) Survival time of neonatal mice under non-suckling condition. Most of the neonatal mice died during 21–24 h after birth under non-suckling condition. The 24-h survivals were immediately killed for experiments. (**d**) Morphological analysis of 3 days' ovaries derived from 20-h non-suckling starvation. (**e**) Quantification of oocyte and follicle numbers in panel (**d**). Data are presented as the mean of three experiments±S.E.M. *P<0.05, **P<0.01

Autophagy was induced in other organs during the early neonatal starvation period.^32^ To investigate whether the role of Atg7 is dependent on autophagy in the neonatal ovaries, we first examined the induction of autophagy by immunostaining the autophagy marker LC3. As shown in Figures 4a and b, the autophagic activity was immediately induced in the neonatal ovaries and reached its maximal level during 3–6 h after birth and then gradually decreased to basal levels at around 24 h. According to our observation, the neonatal mice began suckling at 1–2 h after birth, which is before the maximal autophagic activity time. To confirm that autophagy was induced by neonatal starvation, we separated the neonatal mice from the mother immediately after their birth, which can block their nutrients' supply from milk. Under non-suckling condition, the autophagic activity was upregulated in the neonatal ovaries and reached its maximal level during 3–6 h after birth and then maintained at a relative higher level compared with the normal neonatal mice under suckling condition (Figures 4a and b). In addition, 59% neonatal mice under non-suckling condition died during 21–24 h (Figure 4c); the 24-h survivals were immediately killed for experiments. In order to address whether this non-suckling starvation condition can lead to germ cell loss, survivors under the non-suckling condition were fed at 20 h and lived up to 3 days. As shown in Figures 4d and e, this non-suckling starvation also caused a small-scale oocytes (3156±123.6 *versus* 3620±112.0, *P*<0.05) and follicles (2315±191.6 *versus* 2974±127.4, *P*<0.05) loss compared with the normal group. These results confirmed that autophagy is transiently induced in the ovaries of neonatal mice under physiological conditions.

### Autophagy protects germ cell from over-loss in newborn ovaries

**Figure 5:**
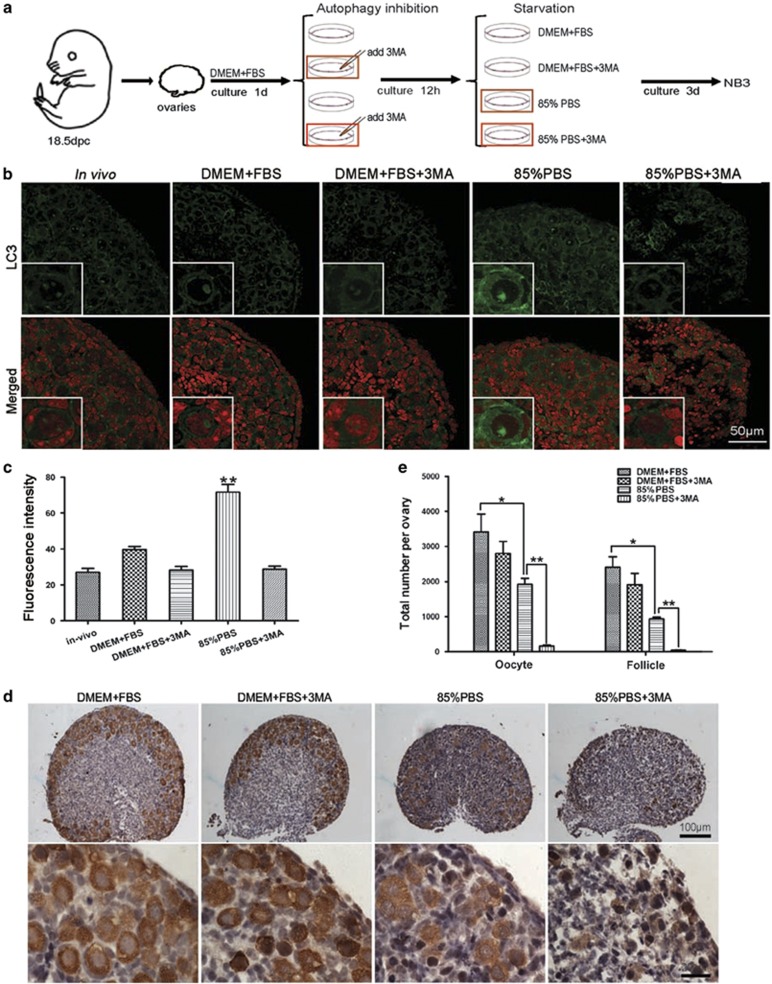
Autophagy protects germ cell from over-loss under starvation condition. (**a**) The diagram of the *in vitro* ovary culture experiments. Dulbecco's modified Eagle's medium+fetal bovine serum (DMEM+FBS) group represented the rich medium. In the 85% phosphate-buffered saline (PBS) group, 85% DMEM+FBS medium were replaced by PBS to mimic the starvation condition. Autophagy was blocked by 3-MA, its final concentration was 5 mM. NB3 represented 3 days after birth. (**b**) Autophagy induction indicated by immunostaining of LC3 in *in vitro* cultured neonatal ovaries. LC3 (in green), nuclear (in red). *In vivo* group represents the ovary derived from the 3-day-old mice. (**c**) Quantification of LC3 fluorescent intensity in panel (**b**). (**d**) Morphological analysis of 3 days *in vitro* cultured ovaries. Germ cell was indicated by immunohistochemical detection of MVH. (**e**) Quantification of oocytes and follicles numbers in 3 days *in vitro* cultured neonatal ovaries. Oocytes surrounded at least by three cells were counted as follicles. Data are presented as the mean of three experiments±S.E.M. *P<0.05, **P<0.01

To address whether Atg7 alone or the whole autophagic machinery is functional in the neonatal mouse ovary, we established an *in vitro* culture system for neonatal ovaries to mimic the *in vivo* neonatal transition process. To accomplish this, ovaries from 18.5-dpc animals were firstly cultured in basic medium^33, 34^ for 24 h, after which the ovaries were treated with autophagy inhibitor 3-methyladenine (3-MA) for autophagy inhibition. Ovaries were starved by incubating in PBS with 15% basic medium. Figure 5a shows the diagram of autophagy inhibition and starvation treatment in the *in vitro* neonatal ovary culture system. After replacing the 85% basic medium with PBS (85% PBS group), the level of LC3 was significantly increased compared with the group under nutrient condition (DMEM+FBS group), which indicated that autophagy was drastically induced in the neonatal ovaries after the nutrient deprivation (Figures 5b and c). In addition, the number of oocytes (1919±170.7) and follicles (939.3±46.94) in the 85% PBS group were largely reduced compared with that in the DMEM+FBS group (3416±510.9, 2407±301.4, *P*<0.05, Figures 5d and e), which implied that the cells were seriously suffering starvation accompanied with cell death. These results suggested that this *in vitro* culture system can mimic the *in vivo* neonatal transition process.

**Figure 6:**
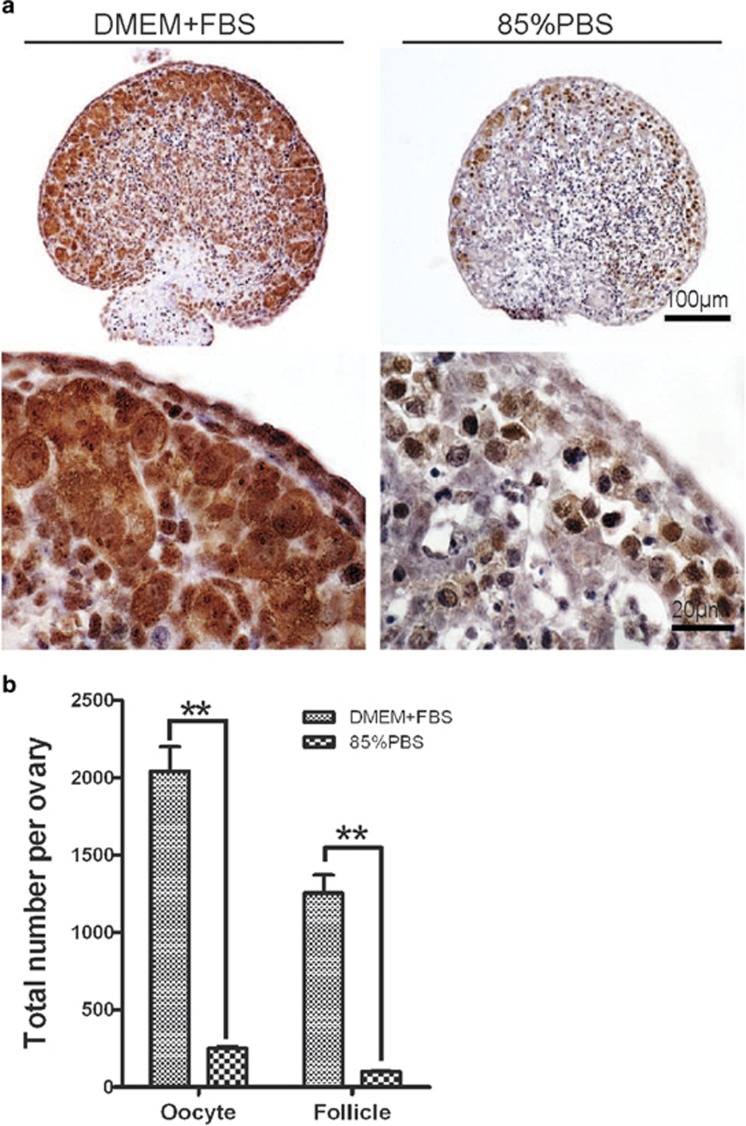
Deletion of *Atg7* causes germ cell over-loss under *in vitro* starvation condition. (**a**) Morphological analysis of *Atg7*^*F/F*^*;Tnap-cre* ovaries cultured for 3 days *in vitro*. (**b**) Quantification of oocyte and follicle numbers in 3 days *in vitro* cultured *Atg7*^*F/F*^*;Tnap-cre* ovaries. Data are presented as the mean of three experiments±S.E.M. **P<0.01

To address the function of autophagy in neonatal mouse ovary, we then blocked autophagy by adding autophagy inhibitor 3-MA into the *in vitro* neonatal ovary culture system. As shown in Figures 5b and c, autophagy was seriously disrupted in the DMEM+FBS+3-MA and 85% PBS+3-MA groups after 3-MA addition. Histomorphological analysis revealed that autophagy inhibition had no significant effect on the oocytes (3416±510.5 *versus* 2801±344.7) and follicles (2407±301.4 *versus* 1907±325.0) under nutrient condition by comparing the DMEM+FBS group to the DMEM+FBS+3-MA group (Figures 5d and e). However, in the 85% PBS+3-MA group, the oocytes (159.3±23.41) and follicles (45.33±6.741) were reduced drastically compared with that in the 85% PBS group (1919.3±170.7, 939.3±46.94, *P*<0.01, Figures 5d and e). Except the number, the morphology and structure of follicles were impaired very dramatically, and typical follicles were rarely found in the 85% PBS+3-MA group (Figure 5d). Furthermore, ovaries derived from *Atg7*^*F/F*^*;Tnap-cre* mice were cultured using the *in vitro* neonatal ovary culture system. As shown in Figures 6a and b, under the starvation condition, the oocytes (251.7±17.43) and follicles (98±12.49) were reduced drastically compared with that in DMEM+FBS group (2041±158.0, 1254±115.3, *P*<0.01), which indicated that deletion of *Atg7* had a similar effect of 3-MA treatment under the starvation condition. These results suggested that it is the whole autophagy machinery rather than Atg7 alone that protects germ cells' over-loss in the neonatal ovaries under the starvation condition.

### Autophagy inhibition leads to oocyte over-loss through apoptosis in the *in vitro* cultured ovaries

**Figure 7:**
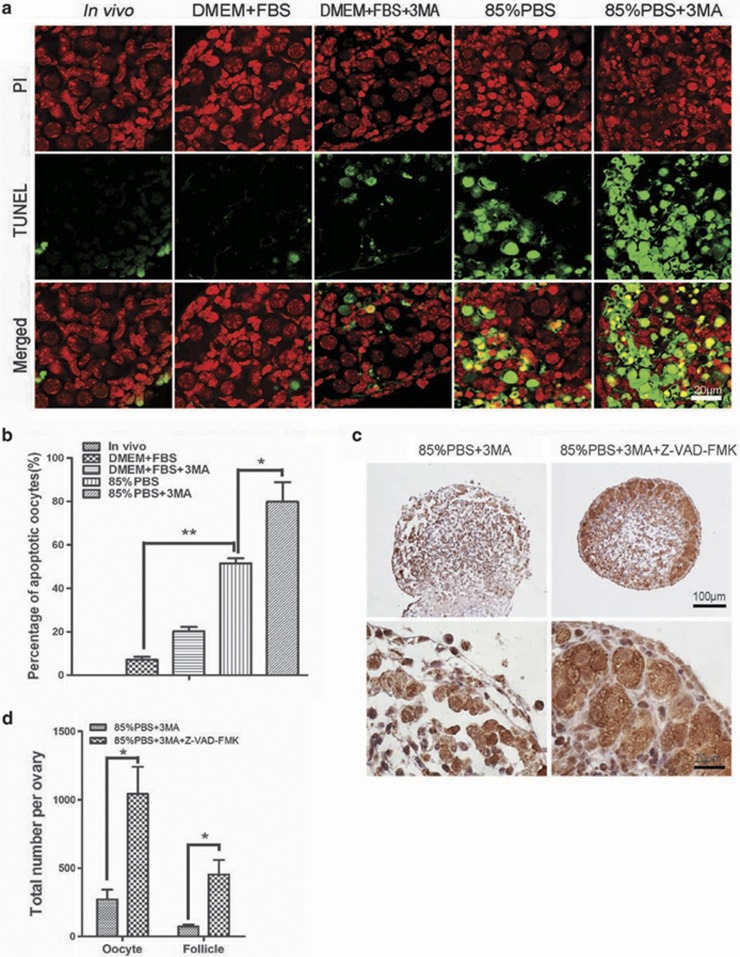
Inhibition of autophagy led to oocytes over-loss through apoptosis under starvation condition. (**a**) *In vitro* cultured ovarian tissues were stained by TUNEL. TUNEL signal (in green), nucleus (in red). (**b**) Percentages of TUNEL-positive oocytes. (**c**) Morphological analysis of 3 days ovaries derived from *in vitro* cultured with or without caspase inhibitor. (**d**) Quantification of the oocyte and follicle numbers in panel (**c**). Data are presented as the mean of three experiments±S.E.M. *P<0.05, **P<0.01

In the neonatal phase in mammals, apoptosis is coupled to autophagy as a means to maintain tissue viability and energy homeostasis in developing tissues.^32, 35, 36^ To test whether inhibition of autophagy lead to germ cell over-loss through apoptosis, we examined the apoptosis process in the *in vitro* cultured neonatal ovaries using the TUNEL (TdT-mediated dUTP nick end labeling) staining. As shown in Figures 7a and b, under the nutrient condition, although the ovaries contained some TUNEL-positive oocytes, there was no significant difference between the DMEM+FBS group and the DMEM+FBS+3-MA group. In accordance with the oocyte/follicle loss results, the percentage of TUNEL-positive oocytes was increased significantly in the 85% PBS group compared with the DMEM+FBS group. Once autophagy was inhibited under starvation condition, the amount of TUNEL-positive oocytes was significantly increased in comparison to the 85% PBS group, about 80% oocytes died by apoptosis. Furthermore, the broad-spectrum caspase inhibitor Z-VAD-FMK (100 *μ*M) was used to block apoptosis in the 85% PBS+3-MA group. Fortunately, the continuous presence of Z-VAD-FMK in the culture medium delayed or inhibited the death of oocytes (Figure 7c), and the number of oocytes (1043±197.2) and follicles (454.7±105.3) were increased drastically compared with that in the 85% PBS+3-MA group (271.7±70.72, 73.33±15.07, *P*<0.05, Figure 7d). These results suggested that inhibition of autophagy lead to oocyte over-loss through apoptosis in the *in vitro* cultured ovaries under starvation condition.

## Discussion

Although a lot of genes have been found to be associated with POI, as a complex disorder, POI may have a number of different genetic etiologies. Actually, defects in the development of PGCs, meiosis, follicle formation, follicular activation and follicular development could all cause POI.^37^ In the present study, we found that germ cell-specific Atg7 knockout led to subfertility in female mice. Similar to POI in human patients, the pool of ovarian follicles decreased dramatically in *Atg7* knockout mice, thus mutations of Atg7 or defects of autophagic machinery might be the etiology of POI.

Further study revealed that oocyte over-loss of *Atg7* knockout mice occurred during the neonatal transition period. The massive germ cell loss shortly after parturition is a mysterious phenomenon in the reproductive biology, and our research might provide a plausible explanation to this phenomenon. In placental mammals, fetus obtains nutrients from the mother through the placenta in prenatal period, which is responsible for the transfer of the bulk of substances between maternal and fetal circulations. However, this trans-placental nutrient supply is suddenly interrupted after birth; neonate must start the arterial supply system. During this transition from fetal to neonatal life, neonates face severe starvation until supply can be restored through milk nutrients.^38^ Most of the organs adapt to this postparturition starvation by inducing autophagy, which can produce amino acids for the maintenance of energy homeostasis by autophagic degradation of ‘self' proteins.^32^ Consistent with a previous report,^34^ we found that autophagy was immediately induced and reached its maximal level during 3–6 h after birth (Figure 4). Inhibition of autophagy by 3-MA in neonatal ovaries cultured *in vitro* led to severe germ cell loss in neonatal ovaries under starvation condition (Figure 5). Hence, germ cells might be sensitive to starvation condition, and the abrupt energy homeostasis shifting during neonatal transition causes massive germ cell loss.

Interestingly, only the ovaries exhibit severe cell loss at birth among the organ systems.^22, 39, 40^ A number of studies have shown that adult stem cells in different tissues have important roles in tissue homeostasis, such as spermatogonial stem cells continuously producing differentiated progeny in testis.^41, 42^ However, the terminally differentiated oocytes are unable to propagate themselves.^43^ Thus only the ovary exhibits profound cell loss at birth among the organ systems. During the neonatal transition, autophagy helps the germ cell to overcome the early neonatal starvation, which can protect germ cell from over-loss in the neonatal ovaries.

The function of autophagy in oocytes during the neonatal transition has been implicated in some previous research; in the spiny mouse, autophagy is found to be involved in follicular atresia and observed in dying oocytes of all follicle types, especially of the primordial and primary ones.^44^ And multiple perinatal mechanisms, including the autophagy process, are found to establish the primordial follicle reserve in murine ovary.^34, 45^ Although it is found that autophagy is not required for egg development in *Drosophila* germline cells, most likely due to the absence of neonatal transition, autophagy still could be induced by starvation in either germline cells or follicle cells in *Drosophila* ovaries.^46^ In mammalian ovaries, the functional role of autophagy in folliculogenesis is limited in germ cells but not granulosa cells, because granulosa cell-specific knockout either *Atg7* (unpublished data) or *Becn1* has no effect on folliculogenesis at all; the granulosa cell-specific *Becn1* ablation results in progesterone production impairment, which led to a subsequent preterm labor phenotype.^47^ Thus, the role of autophagy involved in folliculogenesis is cellular and developmental stage specific.

There are at least two ways to regulate intracellular metabolite supply. One is the acquisition of extracellular nutrients, which can be regulated by growth factor signaling pathways, including the PI3K/Akt system. The other is the activation of intracellular catabolic metabolism to degrade intracellular macromolecules; autophagy is the most extreme form of catabolic pathways.^36^ In general, when growth factors are rich, the growth factor signaling activates the PI3K/Akt pathway and its downstream effectors, mTOR to inhibit autophagy, macromolecular synthesis, ATP production and inhibition of apoptosis.^48, 49, 50^ On the contrary, lacking of growth factors, PI3K/Akt and downstream effectors are inactivated, which can release the inhibition of autophagy by mTOR.^51, 52^ After autophagy induction, cytoplasmic components degraded in autophagosomes/lysosomes provide an alternative source of metabolic substrate to support ATP production and survival. It has been reported that oocyte-specific knockout some of the Pten-PI3K-Akt-Foxo3 pathway-related genes such as Pten and Foxo3 led to global primordial follicle activation.^15, 17^ Oocyte-specific knockout Tsc1 or Tsc2 does not affect the primordial follicle pool development, but the entire pool undergoes global postnatal activation, giving rise to a syndrome of increased follicle depletion and secondary infertility similar to that observed in Pten and Foxo3.^16, 53^ As most of these genes negatively regulate autophagy, the knockout of these genes might activate autophagy in follicles, and autophagy might also participate in primordial follicle activation. Consistent with this postulation, although there are a small amount of primordial follicles in elder Atg7 knockout mice, most of the mice are almost completely infertile, suggesting the dysfunction of these follicles. So autophagy may work as a double-edged sword in oocytes or follicles: too much results in primordial follicle activation and ends up with POI; too low results in oocytes over-loss, which also ends up with POI. Thus autophagic level needs to be well maintained in the ovary to retain female fertility.

Taken together, our present study demonstrated that Atg7 had critical roles in ovarian reserve of primordial follicles, and germ cell-specific Atg7 knockout resulted in reproductive defects with serious follicle loss in female mice. Further investigation revealed that disruption of autophagy was responsible for this follicle loss. After Atg7 depletion, the germ cells lost protection from autophagy to overcome the neonatal starvation, which led to poor ovarian reserve of primordial follicles. In conclusion, our research highlights new mechanisms for the regulation of ovarian reserve of primordial follicles and potential pathological cause of POI in human patients.

## Materials and Methods

### Mice

The *Atg7*^*F/F*^ mouse strain (RBRC02759) was purchased from the RIKEN BioResource Center (Tokyo, Japan). *Atg7*^*F/F*^; *Tnap-Cre* mice were bred from *Atg7*^*F/F*^ mice and *Tnap-Cre* mice, which were purchased from the Jackson Laboratory (Sacramento, CA, USA, 008569). The mice were bred under controlled environmental conditions with free access, illumination was provided between 0800 and 2000 hours. All animal experiments were approved by the Animal Research Panel of the Committee on Research Practice of the University of Chinese Academy of Sciences.

### Tissue collection and histological analysis

The ovaries were dissected immediately following euthanasia. Then they were fixed in 4% paraformaldehyde (pH 7.5) overnight at 4 °C, dehydrated, and embedded in paraffin, after which sections (5-*μ*m thickness) were cut and mounted on glass slides. Following deparaffinization, the slides were stained with hematoxylin and eosin for histological analysis.

### Quantification of ovarian follicles

To count the numbers of follicles, paraffin-embedded ovaries were serially sectioned at 8-*μ*m thickness and every fifth section was mounted on slides as previously described by Tilly^54^ in 2003. Ovarian follicles at different developmental stages were counted in collected sections of an ovary, based on the well-accepted standards established by Pedersen and Peters.^55^ Repetitive counting of the same follicles in every fifth section through the entire ovary was avoided by counting only follicles containing oocytes with a visible nucleolus.

### Ovarian tissue isolation and *in vitro* culturing

Briefly, fetal mouse ovaries of 18.5 dpc were obtained from pregnant CD-1 mice, and the morning when copulation plug was observed was designated as the 0.5 dpc. The fetal ovaries, without attached mesonephroses, were selected and cultured in 500 *μ*l basic medium (DMEM/F12, 10% FBS (Gibco, Carlsbad, CA, USA), 1% ITS (Sigma, St. Louis, MO, USA), 1% penicillin/streptomycin (Sigma)) in a 24-well plate. Ovaries on 24-well plates were cultured at 37 °C in modular incubation chambers, thoroughly infused with a gas mixture of 5% CO_2_ and air. The next day, ovarian tissues were treated with the autophagy inhibitor 3-MA (Sigma, M9281), at a concentration of 5 mM. After 12-h 3-MA treatment, the medium was changed to starvation condition with replacement of 85% medium by PBS. The day at which isolated ovaries were cultured in starvation condition was defined as Day 0. Experiments were repeated at least three times. We used at least three ovaries each time for different groups. For apoptosis inhibition, caspase inhibitor Z-VAD-FMK (Beyotime Institute of Biotechnology, Haimen, China, C1202) was used at a concentration of 100 uM.

### Immunofluorescence

Ovaries were immediately embedded in an optimum cutting temperature compound and cut in 8-μm sections using a microtome-cryostat. Sections were fixed with 4% paraformaldehyde and rinsed in PBS three times (pH 7.4), treated with 0.1% Triton X-100 for 10 min, rinsed in PBS three times, blocked in blocking buffer (3% BSA, 10% normal goat serum in PBS) for 30 min and then incubated with rabbit anti-LC3 polyclonal antibody (Abcam, Cambridge, UK, ab58610) at 4 °C overnight. After three rinses in PBS, the sections were incubated with goat anti-rabbit IgG conjugated with fluorescein isothiocyanate at a dilution of 1 : 200 (Zhong Shan Golden Bridge Biotechnology, Beijing, China, ZF-0311) for 30 min at 37 °C. Propidium iodide (PI) was used to label nuclei. Images were taken immediately using a LSM 780/710 microscope (Zeiss, Oberkochen, Germany).

### Immunohistochemistry

Ovaries used for histological analysis were collected from *in vitro* cultured ovaries, then fixed in 4% paraformaldehyde overnight at 4 °C, dehydrated and embedded in paraffin. The samples were serially sectioned at 5 *μ*m as previously described by Tilly^54^ in 2003. Paraffin sections were fixed with 4% paraformaldehyde and rinsed in PBS three times. Then sections were boiled for 15 min in sodium citrate buffer for antigen retrieval. After blocked in blocking buffer (3% BSA, 10% normal goat serum in PBS) for 30 min, sections were incubated with rabbit anti-MVH polyclonal antibody (Abcam, ab13840) at 4 °C overnight, followed by staining with the HRP-conjugated secondary antibody. Finally, the sections were stained with DAB (3, 3′-diaminobenzidine), and the nuclei were stained with hematoxylin. To count the numbers of oocytes and follicles, every third section was mounted on slides.

### TUNEL

TUNEL assays were carried out with the *In Situ* Cell Death Detection Kit (Roche Diagnostics, Basel, Switzerland, 11684795910) according to the manufacturer's instructions. Briefly, sections of ovaries were heated at 60 °C for 2 h followed by washing in xylene and rehydration through a graded series of ethanol and double distilled water. Then sections were treated with proteinase K for 15 min at room temperature and rinsed twice with PBS. After the TUNEL reaction mixture was added, slides were incubated in a humidified atmosphere for 60 min at 37 °C in the dark. Finally, the sections were stained with PI.

### Statistical analysis

All experiments were repeated at least three times, representing the mean±S.E.M., within an individual experiment. The differences between the treatment and control groups were analyzed by ANOVA, and differences were calculated by the Tukey's test. The data were considered significant when the *P*-value was <0.05 (*) or <0.01 (**).

## Abbreviations

Atg7: autophagy-related gene 7
LC3: microtubule-associated protein 1 light chain 3
POI: primary ovarian insufficiency
MVH: mouse vasa homolog
3-MA: 3-methyladenine
TUNEL: TdT-mediated dUTP nick end labeling
dpc: day postcoitum
PI: propidium iodide
